# Unjust Treatment of Physicians by the State

**Published:** 1890-11

**Authors:** James Johnston

**Affiliations:** Brownwood


### Correspondence.

For Daniel's Texas Medical Journal.

### UNJUST TREATMENT BY THE STATE.

### A Texas Soldier Undertakes to Regulate Fees for the Practice of Medicine in Texas.

During the fence-cutting excitement—about six years ago—eight Texas rangers, under the command of Capt. Scott, were camped about two and a half miles from Brownwood. In the early spring measles broke out in the camp and one severe case of pheumonia. The weather was damp and cold, and the Rangers were living in tents with very scant bed clothes and very poorly provided for. Five of the men suffered with measles, and one who has been through the late war knows something about the fatality of "camp measles." I was engaged by Capt. Scott to attend to the sick, with directions to give them the necessary medical attention and provide everything necessary for their comfort and safety, with the assurance that the State would pay for it. So I went to work in good faith, secured the medicine from the druggist, and even secured a post-auger, at an expense of $3.50, for the purpose of sinking posts to prevent the tents from being blown down and for repairing pens for the horses. I made from one to two vistis per day; some of the patients were very sick; in one case I had to use the catheter twice a day for over a week. I gave the same attention, and charged exactly the same fees as in private practice. When all the patients had recovered I made out my account as directed by Capt. Scott, had the account sworn to before a notary and endorsed as correct by Capt. Scott, and forwarded to Austin for collection; but in the course of a few days Captain Scott informed me that Adjutant General King refused to pay the bill, on the ground that I was only entitled to a fee for waiting on one patient, although I had waited on as many as five sick (and used the catheter with one) during one visit. He also objected to the druggist's bill (which I was bound for) on the ground that one prescription cost over a dollar. I wrote the Adjutant-General, asking him to submit my account to any regular physician in Austin, and the drug account to any druggist, and I would submit to their decision,—but he refused, and when I asked Captain Scott why he objected to such a fair proposition, his answer was he did not know, except that he was "hard-headed" and thought he knew best himself.

As I was brought up under a government where there is redress for every wrong, I thought it was so here; so I determined on bringing the case into court, but soon found that I could not sue the State, without first obtaining permission from the legislature to do so. My representative brought the case before that august body, but in accordance with the ordinary manner of treating with contempt any request coming from the medical profession, and, no doubt, believing that doctors are fast growing rich from the large fees extorted from their patients, refused to allow the State to be sued.

So I was wronged out of my just earnings. The distance was two and a half miles, the livery bill $2 each trip, leaving 50 cents for waiting on as many as five patients at a time. The fees I charged were $2 for each patient waited on, which is about the general custom all over the State. Now, if the General's views are correct, the rule would hold good if 500 patients, or 5000 required attention, the physician would have 50 cents net for all his work; but in my case, when I paid the druggist the full amount of his bill and the hardware man for the post auger, and other bills for stimulants and cordials, my fifty cents per visit was exhausted.

I do not report this case as a complaint against Adjutant-General King, but to show to the medical profession of this State that there must be something wrong in the government when a man can be deprived of his property or wages, without a due process of law. I refer to article xiv, section 1, amended Constitution of the United States. JAMES JOHNSTON, M. D.

BROWNWOOD, Oct. 1, 1890.

